# Supplementary material for: Modeling glioblastoma heterogeneity as a dynamic network of cell states
Source: Mol Syst Biol. 2021 Sep 16;17(9):e10105. doi: 10.15252/msb.202010105 (PMC8444284; doi:10.15252/msb.202010105)
Supplement: Supplementary file 6 — Source Data for Figure 5 [file MSB-17-e10105-s004.zip › Figure5A_sourcedata/GSEA_3071/hallmarks_stateI.GseaPreranked.1623742531165/HALLMARK_TGF_BETA_SIGNALING.html]

Details for gene set HALLMARK\_TGF\_BETA\_SIGNALING[GSEA]

|  || Dataset | state13071 |
| Phenotype | NoPhenotypeAvailable |
| Upregulated in class | na\_pos |
| GeneSet | HALLMARK\_TGF\_BETA\_SIGNALING |
| Enrichment Score (ES) | 0.4108355 |
| Normalized Enrichment Score (NES) | 1.6790715 |
| Nominal p-value | 0.033333335 |
| FDR q-value | 0.025866024 |
| FWER p-Value | 0.239 |
Table: GSEA Results Summary

  

Fig 1: Enrichment plot: HALLMARK\_TGF\_BETA\_SIGNALING      
 Profile of the Running ES Score & Positions of GeneSet Members on the Rank Ordered List

  

| PROBE | GENE SYMBOL | GENE\_TITLE | RANK IN GENE LIST | RANK METRIC SCORE | RUNNING ES | CORE ENRICHMENT || 1 | ID3 |  |  | 86 | 1.152 | 0.0712 | Yes |
| 2 | SLC20A1 |  |  | 116 | 1.035 | 0.1491 | Yes |
| 3 | TGFB1 |  |  | 165 | 0.884 | 0.2090 | Yes |
| 4 | TGFBR1 |  |  | 224 | 0.778 | 0.2572 | Yes |
| 5 | BCAR3 |  |  | 354 | 0.628 | 0.2725 | Yes |
| 6 | JUNB |  |  | 454 | 0.546 | 0.2896 | Yes |
| 7 | TJP1 |  |  | 557 | 0.477 | 0.3001 | Yes |
| 8 | SMAD7 |  |  | 597 | 0.454 | 0.3268 | Yes |
| 9 | THBS1 |  |  | 613 | 0.446 | 0.3596 | Yes |
| 10 | FURIN |  |  | 663 | 0.426 | 0.3810 | Yes |
| 11 | ID1 |  |  | 682 | 0.420 | 0.4108 | Yes |
| 12 | ACVR1 |  |  | 1317 | 0.265 | 0.2513 | No |
| 13 | CDKN1C |  |  | 1571 | -0.264 | 0.2007 | No |
| 14 | RHOA |  |  | 1843 | -0.298 | 0.1479 | No |
| 15 | TGIF1 |  |  | 1955 | -0.313 | 0.1422 | No |
| 16 | SMURF2 |  |  | 1982 | -0.316 | 0.1610 | No |
| 17 | PMEPA1 |  |  | 2107 | -0.336 | 0.1534 | No |
| 18 | SMAD1 |  |  | 2265 | -0.365 | 0.1388 | No |
| 19 | APC |  |  | 2550 | -0.427 | 0.0930 | No |
| 20 | BMP2 |  |  | 2589 | -0.436 | 0.1184 | No |
| 21 | SERPINE1 |  |  | 3050 | -0.634 | 0.0394 | No |
| 22 | ID2 |  |  | 3374 | -1.110 | 0.0393 | No |
Table: GSEA details [plain text format]

  

Fig 2: HALLMARK\_TGF\_BETA\_SIGNALING: Random ES distribution      
 Gene set null distribution of ES for **HALLMARK\_TGF\_BETA\_SIGNALING**
